# Understanding tumor ecosystems by single-cell sequencing: promises and limitations

**DOI:** 10.1186/s13059-018-1593-z

**Published:** 2018-12-03

**Authors:** Xianwen Ren, Boxi Kang, Zemin Zhang

**Affiliations:** Beijing Advanced Innovation Centre for Genomics, Peking-Tsinghua Centre for Life Sciences, Biomedical Pioneering Innovation Center (BIOPIC), School of Life Sciences, Peking University, Beijing, 100871 China

## Abstract

Cellular heterogeneity within and across tumors has been a major obstacle in understanding and treating cancer, and the complex heterogeneity is masked if bulk tumor tissues are used for analysis. The advent of rapidly developing single-cell sequencing technologies, which include methods related to single-cell genome, epigenome, transcriptome, and multi-omics sequencing, have been applied to cancer research and led to exciting new findings in the fields of cancer evolution, metastasis, resistance to therapy, and tumor microenvironment. In this review, we discuss recent advances and limitations of these new technologies and their potential applications in cancer studies.

## Introduction

A single cell is the ultimate unit of life activity, in which genetic mechanisms and the cellular environment interplay with each other and shape the formation and function of such complex structures as tissues and organs. Dissecting the composition and characterizing the interaction, dynamics, and function at the single-cell resolution are crucial for fully understanding the biology of almost all life phenomena, under both normal and diseased conditions. Cancer, a disease caused by somatic mutations conferring uncontrolled proliferation and invasiveness, could in particular benefit from advances in single-cell analysis. During oncogenesis, different populations of cancer cells that are genetically heterogeneous emerge, evolve, and interact with cells in the tumor microenvironment, which leads to host metabolism hijacking, immune evasion, metastasis to other body parts, and eventual mortality. Cancer cells can also manifest resistance to various therapeutic drugs through cellular heterogeneity and plasticity. Cancer is increasingly viewed as a ‘tumor ecosystem’, a community in which tumor cells cooperate with other tumor cells and host cells in their microenvironment, and can also adapt and evolve to changing conditions [1–5].

Detailed understanding of tumor ecosystems at single-cell resolution has been limited for technological reasons. Conventional genomic, transcriptomic, and epigenomic sequencing protocols require microgram-level input materials, and so cancer-related genomic studies were largely limited to bulk tumor sequencing, which does not address intratumor heterogeneity and complexity. The advent of single-cell sequencing technologies [6–8] has shifted cancer research to a new paradigm and revolutionized our understanding of cancer evolution [7–22], tumor heterogeneity [23–46], and the tumor microenvironment [47–59]. Development of single-cell sequencing technologies and the applications in cancer research have been astonishing in the past decade, but many challenges still exist and much remains to be explored. Single-cell cancer genomic studies have been reviewed previously [60–63]. In this review, we summarize recent progress and limitations in cancer sample single-cell sequencing with a focus on the dissection of tumor ecosystems.

## Overview of single-cell sequencing and analysis

**Fig. 1:**
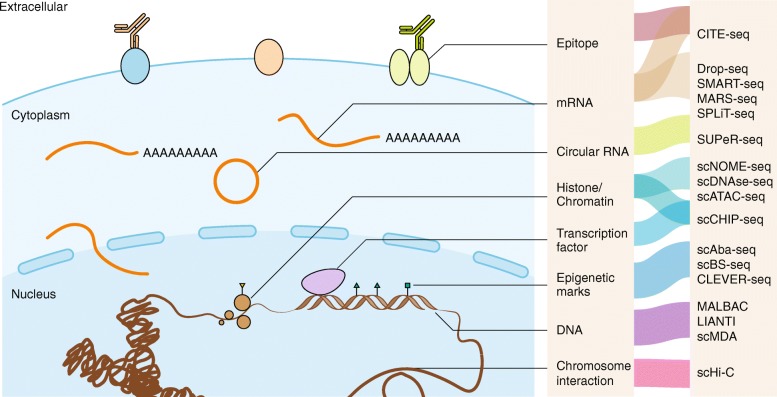
State of the art of single-cell sequencing technologies. Single-cell sequencing technologies have been designed for almost all the molecular layers of genetic information flow from DNA to RNA and proteins. For each molecular layer, multiple technologies have been developed, all of which have specific advantages and disadvantages. Single-cell multi-omic technologies are close to comprehensively depicting the state of the same cells. We apologize for the exclusion of many single-cell sequencing methods due to the limited figure space

Single-cell sequencing technologies have improved considerably from the initial proof-of-principle studies [6–8]. Modification of the underlying molecular biology and chemistry of single-cell library preparation has provided diverse approaches to obtain and amplify single-cell nucleic acids for subsequent high-throughput sequencing [64–72] (Fig. 1). Because an individual cancer cell typically contains only ∼6–12 pg of DNA and 10–50 pg of total RNA (depending on the cell types and status) [73], amplification is essential for single-cell library preparation to fulfill the sequencing input requirements, although both false positive and false negative errors may arise in the process [74]. Single-cell DNA and RNA sequencing, epigenomic sequencing [68, 70, 72, 75], and simultaneous sequencing of the genome, transcriptome, epigenome, and epitopes of the same single cell [32, 35, 76–80] are all now possible, and can facilitate exploration of the connection between cellular genotypes to phenotypes. Furthermore, the throughput of single-cell sequencing technologies has improved vastly, with some methods allowing simultaneous sequencing of tens of thousands of single cells in one run [81–86]. Methods that couple additional experimental techniques with single-cell sequencing technologies are also gaining traction [21, 87–91], to provide a more integrated analysis of single cells.

Accompanying the tremendous progress of experimental single-cell sequencing technologies, specialized bioinformatics and algorithmic approaches have also been developed to best interpret the single-cell data while reducing their technological noise. Examples of these approaches include the imputation of dropout events [92–95], normalization and correction of batch effects [96–100], clustering for identification of cell types [98, 101–108], pseudo-temporal trajectory inference [109–112], spatial position inference [87, 88, 90], and data visualization [102, 113–115]. Progress in this area requires the application of statistics, probability theory, and computing technologies, which lead to new algorithms, software packages, databases, and web servers. Detailed information of specific single-cell technologies and the underlying principles of the algorithms have been elegantly discussed in other reviews [61, 64–70, 72, 116–123]. This myriad of experimental and computational methods is becoming the new foundation for uncovering the mystery of cancer complexity at the single-cell resolution.

Despite the dramatic advances, substantial limitations and challenges still exist in single-cell sequencing. The first challenge lies in the technological noise introduced during the amplification step. Notable allelic dropouts (i.e., amplification and sequencing of only one allele of a particular gene in a diploid/multiploid cell) and non-uniform genome coverage hinder the accurate detection of single nucleotide variants (SNVs) at the genome or exome level. These problems can be partially alleviated by the LIANTI (linear amplification via transposon insertion) method [40], which implements a linear genomic amplification by bacterial transposons and reportedly reaches improvements in genome coverage (~ 97%), allelic dropout rates (< 0.19) and false negative rates (< 0.47). Similarly, in single-cell RNA sequencing (scRNA-seq), lowly expressed genes are prone to dropout and susceptible to technological noise even when detected, although they often encode proteins with important regulatory or signaling functions. These technological issues are more profound for scRNA-seq technologies designed to offer higher throughput [81, 84]. Although many computational methods are available to model or impute dropout events [92, 94, 95], their performances vary and may introduce artificial biases. Much effort is needed to fully address this challenge.

The second challenge is that only a small fraction of cells from bulk tissues can be sequenced. Bulk tissues consist of millions of cells, but present studies can often only sequence hundreds to thousands of single cells because of technological and economic limitations [9–11, 20, 25, 124–126]. To what extent the sequenced cells represent the distribution of cells in the entire tissue of interest is not clear. A plausible solution to address this challenge would be to further improve the throughput of cellular captures, e.g., MARS-seq [82] and SPLiT-seq [86], or alternatively to combine bulk and single-cell sequencing together and then conduct deconvolution analysis [127]. Deconvolution analysis for bulk RNA-seq data uses cell-type signature genes as inputs [128–130], which can be substituted by single-cell sequencing results, although critical computational challenges still exist, such as collinearity among single cells. If marker genes for known cell types are orthogonal to each other, the proportions of each cell type in a bulk sample can be reliably estimated. However, collinearity of gene expression exists widely among single cells, which complicates the deconvolution process. At present, successful deconvolution of bulk RNA-seq data based on scRNA-seq-defined signatures has been reported only in cases where orthogonal molecular signatures and fine cluster structures are well balanced [131]. The wide usage of scRNA-seq based deconvolution will hinge upon the availability of comprehensive single-cell clusters and the development of general methods for selecting orthogonal signatures for each cell type.

Spatial information of single cells in the tissue is often lost during the isolation step and thus single-cell sequencing data typically do not show how cells are organized to implement the concerted function within a tissue of interest. Many new techniques have been developed to keep or restore the spatial information of sequenced single cells such as fluorescence in situ hybridization (FISH), single-molecule fluorescence in situ hybridization (smFISH), laser capture microdissection, laser scanning microscopy, including two-photon laser scanning microscopy, and fluorescence in situ sequencing [21, 30, 87–91, 132–143]. However, at present all of these techniques have inherent limitations and only apply to specific spatial architecture. For example, while FISH-based technologies can map the spatial distribution of a set of selected genes upon which the spatial information of single cells subject to RNA-seq can be reconstructed via probabilistic inference, the methods are limited to two dimensions and the inference is primarily dependent on the availability of marker genes that can properly discriminate the spatial characteristics with sufficient resolutions. Other conditions for valid marker genes include accurate and robust estimation of their expression levels, but this requirement can be greatly compromised by inherent dropout in scRNA-seq protocols. Accurate restoration of single cell spatial positions via FISH-based inference also requires replicable tissues for parallel FISH and scRNA-seq, which can be only approximately fulfilled on model organisms. For human cancers, however, such requirements usually cannot be met and spatial-recording methods have thus been proposed. With laser capture microdissection, single cells are obtained simultaneously when their spatial information is recorded. However, the cellular throughput of such methods is extremely limited due to operation difficulties, and the biological interpretation of the recorded spatial positions are confined because adjacent cells cannot be properly dissected for scRNA-seq, whereas sequenced cells are often distantly distributed. Low molecular throughput is also problematic with these recently developed in situ sequencing methods. Typically, only tens or hundreds of known genes can be in situ labeled or sequenced, far from the requirement of fully understanding the molecular landscapes of single cells of interest. Furthermore, the replicability of such complicated experiments also imposes barriers for their practical applications to human samples.

Because single-cell sequencing captures individual cells at a particular time point, other factors such as cell cycle and functional state must be considered. By contrast, these factors are often ignored in bulk sequencing due to the average effect. Cell cycle phases can be discerned by phase-specific expression analysis [144–146], but cell types and cell states can be hard to distinguish. Sometimes even cancer cells cannot be easily distinguished from normal cells, although inferred DNA copy numbers are often used for this purpose [22, 47, 51]. More robust methods are needed for cell type determination in silico.

Compared to traditional bulk sequencing technologies, which characterize samples via a gene-by-sample matrix, single-cell sequencing adds a cellular layer between genes and samples, which results in a gene-by-cell-by-sample data structure. Addition of the cellular dimension allows simultaneous characterization of samples at both the molecular and cellular level. However, bioinformatics and algorithmic methods for single-cell sequencing data analysis are generally developed for gene-by-cell data, which essentially have the same structure with the gene-by-sample matrices. Although methods exploiting the cellular dimension for phenotype classification have been proposed [147], tools sufficiently employing all the molecular, cellular, and sample information of the new data structure are still needed.

Given the maturation of single-cell sequencing technologies, especially scRNA-seq, the scale of datasets of one study soon increases from hundreds to tens of thousands and even millions of cells. For large programs, e.g., the Human Cell Atlas project [148], the volume of data demands more robust computer hardware and software. Although a few down-sampling or convolution-based methods have been proposed to manage large-scale scRNA-seq data for clustering and differential expression analysis [149–151], efficient and effective algorithms are of pressing need to circumvent these difficulties.

## Complexity of tumor ecosystems

**Fig. 2:**
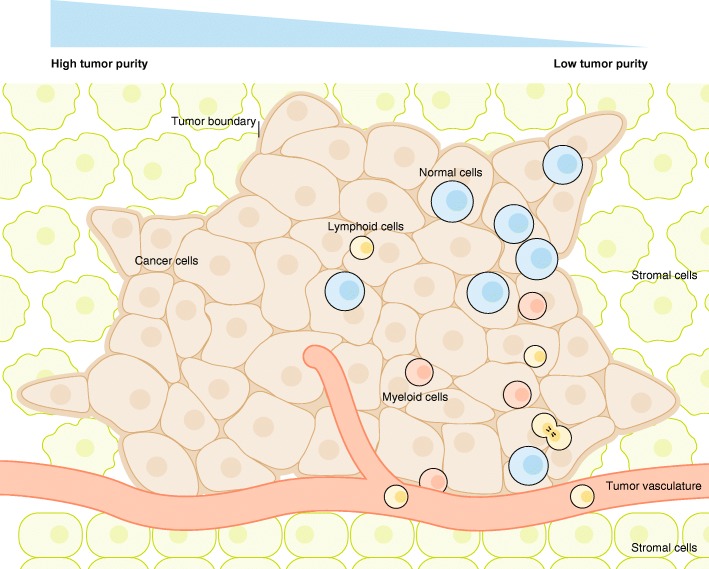
Spatial heterogeneity of tumors. A tumor is a complex ecosystem composed of various cell types which show heterogeneous spatial distributions. The cell types within a tumor generally contain cancer cell clones, normal cells that have not been transformed, stromal cells, immune cells, and endothelial cells. Because of the spatial heterogeneity, bulk sequencing from a specific specimen will produce an average signal of thousands of cells with unknown composition, which forms a hidden confounding factor that interferes with the interpretations of cancer research and diagnosis. Single-cell sequencing inherently has the power to dissect the cellular composition of tissues, providing a powerful tool to advance cancer studies

Cancer is known for its heterogeneity, at the inter- and intra-tumor levels [152]. Within a tumor, different spatial sites have different composition of cancer cell clones (Fig. 2), which results in spatial heterogeneity [152]. As cancer cells evolve, temporal variations also arise during the course of cancer genesis and progression, causing temporal heterogeneity [152]. In addition to cancer cells, tumors are also infiltrated with stromal, immune, and other cell types. The diversity of these cells forms the basis of the heterogeneity of the tumor microenvironments [1, 4, 153]. The complex and dynamic nature of cancer heterogeneity within tumors is analogous to ecosystems. Thorough understanding of the composition, interactions, dynamics, and operating principles of tumor ecosystems is key to understanding cancer evolution and the emergence of drug resistance. Multi-region sampling coupled with bulk sequencing is a plausible approach to investigating intra-tumor heterogeneity on the genome scale [36, 154, 155]. However, although this approach reveals intra-tumor heterogeneity, it cannot directly dissect the cellular composition of tumors. Computational deconvolution techniques could help infer the cellular composition of tumors, but such analyses are limited to a few known cell types [128–130]. Single-cell sequencing represents a quantum technological leap, as it allows the most precise dissection of the complex architecture of tumors while capturing rare cell types. Here, we review recent progress on understanding tumor ecosystems using single-cell sequencing technologies (Table 1).

**Table 1:** Recent progress of cancer studies based on single-cell sequencing

Technology	Study topic	References
Single-cell DNA-seq	Heterogeneity of cancer clones	[18, 33, 40, 45, 157]
Single-cell DNA-seq	Mutation profiles of CTCs	[16, 43]
Single-cell RNA-seq	Expression patterns of cancer cells upon treatment	[19, 29, 46, 165]
Single-cell RNA-seq	Expression heterogeneity and dynamics of cancer cells	[22, 37, 38, 41, 44]
Single-cell RNA-seq	Expression patterns of CTCs	[31]
Single-cell RNA-seq	Heterogeneity of tumor microenvironment	[47–52, 55–59]
Single-cell RNA-seq, mass cytometry	Heterogeneity of tumor microenvironment	[53, 54]
Single-cell DNA-seq and RNA-seq	Integrated analysis of cancer cells	[20, 166]
Single-cell epigenomics	Epigenomics of cancer cells	[187]
Single-cell multi-omics	Multi-omics analyses of the same cancer cells	[32]
Single-cell-derived organoids	Diversification of cancer cells	[42]
Spatial single-cell sequencing	Spatial heterogeneity and metastasis of cancer cells	[21, 30]
Single-cell DNA-seq	Amplification methods	[7, 8, 39, 40, 172, 206–208]

## Decomposition of clonal and sub-clonal tumor structure

Early success of single-cell sequencing applications in cancer research came from the studies of clonal and sub-clonal structure of primary tumors. DNA-based single-cell sequencing has been applied to breast [7, 20, 21, 26, 156, 157], kidney [158], bladder [159], and colon tumors [39, 160, 161], glioblastoma [162], and hematological malignancies such as acute myeloid leukemia and acute lymphoblastic leukemia [11, 33, 163–165]. These studies demonstrated the existence of common mutations among different cancer cell clones in individual cancer patients, which provided evidence for the origin of common cancerous cells and subsequent clonal evolution. Meanwhile, the application of scRNA-seq in glioma [22, 51, 166] demonstrated that cell differentiation of neural stem cells also contributes to tumor heterogeneity, thus supporting a cancer stem cell model. Notably, a recent study of intra-tumor diversification of colorectal cancers [42] integrated single-cell technologies and tumor organoid culture to show that cancer cells had several times more somatic mutations than normal cells. The authors of this study also observed that most of the mutations occurred during the final dominant clonal expansion, contributed by mutational processes absent from normal controls. In addition to canonical mutations, transcriptomic alterations and DNA methylation were cell-autonomous, stable, and followed the phylogenetic tree of each cancer. The study by Roerink et al. [42] provided a paradigm of cancer evolution by characterizing clonal and sub-clonal tumor structures, and indicated the potential dynamics of cancer progression. These findings exemplify the unique power of single-cell sequencing to characterize the diversity of cancer cells, resulting in different evolutionary models between cancers. In particular, single-cell data challenged the cancer stem cell model by showing that continued proliferation and clonal expansion formed the majority of tumor cells. Furthermore, scRNA-seq data supported the cancer stem cell model by demonstrating the contribution of cell differentiation to tumor heterogeneity. Copy number alternations (CNAs) and point mutations of cancer cells were subject to different evolutionary modes, with the former preferring punctuated evolution and the latter preferring gradual accumulation. Outstanding disparities need to be resolved before consistent models of cancer genesis and evolution can be applied to a wide range of cancers. Studies with larger sample size and higher molecular and cellular resolution are needed to reconcile various cancer evolution models. Sequencing analysis of single-cell-derived organoids could provide a template for investigating cancer evolution, but this should be extended to larger samples and other cancer types.

## Monitoring cancer progress through characterization of circulating tumor cells

Circulating tumor cells (CTCs) are extremely rare in blood (1 in 10^6^), with only tens of cells captured from a typical blood draw [60]. The application of bulk sequencing to such limited input material for genomic exploration is difficult, hindering the analysis of cancer cell migration via blood. Single-cell sequencing has transformed the ability to characterize CTCs and has been used to identify metastatic potential of CTCs in cancer metastasis models, to monitor abnormal signaling pathways for drug-resistance prediction. By characterizing mutation profiles of CTCs, their tissue sources can be matched to the positions of primary and metastatic tumors [13, 16, 24, 167, 168]. This type of analysis holds great potential in early cancer detection and real-time monitoring of disease progression with or without treatment. Furthermore, the origin and destination of CTCs could be further explored to reveal the dissemination conditions of specific tumors. The application of DNA-based single-cell sequencing to CTCs in colon cancer [161], melanoma [169], lung cancer [170], and prostate cancer [171, 172] revealed that the copy number profiles of CTCs are highly similar to primary and metastatic tumors but point mutation profiles show much greater variations, consistent with punctuated evolution of CNAs and gradual evolution of point mutations observed within tumors. A recent integrative analysis of colon, breast, gastric, and prostate cancers by single-cell DNA sequencing compared the mutation profiles between primary tumor cells and CTCs, and revealed convergent evolution of CNAs from primary cancer tissues to CTCs [16]. Remarkably, CNAs affecting the oncogene *MYC* and the tumor suppressor gene *PTEN* were observed only in a minor proportion of primary tumor cells but were present in all CTCs spanning multiple cancer types. These observations suggest that the potential of primary tumor cells to transit into CTCs are quite uneven, or otherwise strong selection pressure exists upon CTCs during the metastasis process. To resolve the detailed molecular mechanisms involved in the generation of CTCs in primary tumors to colonization in metastasis sites, it will be important to temporally trace the variations of CTCs during cancer progression from primary tumors to metastasis in both a research and clinical setting. Furthermore, scRNA-seq has been used in the study of CTCs in melanoma [173], breast [167], pancreatic [126, 174], and prostate cancers [31], revealing specific transcriptional signatures of CTCs relative to their primary and metastatic tumors. Extracellular matrix proteins were specifically expressed by CTCs, and plakoglobin appeared to be a key regulator of CTC clusters with survival advantages distinct from individual CTCs. Furthermore, abnormal signaling pathways for drug resistance prediction can be monitored using scRNA-seq of CTCs, as illustrated by the Miyamoto et al. study [31], in which scRNA-Seq profiling of 77 CTCs from 13 prostate cancer patients revealed extensive heterogeneity of the androgen receptor gene at both expression and splicing levels. Activation of non-canonical Wnt signaling was observed in the retrospective study of CTCs from patients treated with an androgen receptor inhibitor, indicating the potential resistance to therapy. Despite enviable progress, CTC studies remain limited by difficulties in the detection and enrichment of CTCs from blood. How to effectively obtain insight into the generation, progress, metastasis, and response to therapies of the entire tumor through the characterization of CTCs is still an elusive question.

## Interrogating the genesis and evolution of therapy resistance

Chemotherapy and targeted therapies have been important weapons to combat cancers, but drug resistance is common for most tumors. Due to the complexity of cancer drug resistance, the underlying mechanisms remain poorly understood for most human cancers, which hampers the development of new approaches to overcome drug resistance. An important question to address is whether drug resistance arises from rare pre-existing subclones with drug-resistant phenotypes prior to treatment (intrinsic resistance) or, alternatively, is acquired through induction of new mutations conferring drug-resistance (acquired resistance). Acquired versus intrinsic resistance has been studied for decades in bacteria, which are single-cell systems [175], but remains elusive in most human cancers. Single-cell sequencing can be used to resolve tumor heterogeneity, reconstruct the evolutionary trajectories of cancer cells, and identify rare subclones, and has therefore been a promising method to address drug resistance [19, 25, 29, 47, 165]. The recent study by Kim et al. [20] of triple-negative breast cancers treated with neoadjuvant chemotherapy employed both single-cell DNA- and RNA-sequencing to resolve the genesis and evolution of drug-resistant clones. Using DNA data from 900 cells and RNA data from 6862 cells, CNAs in drug-resistant subclones were found to be pre-existing and adaptively selected while their expression profiles were acquired through transcriptional reprogramming in response to chemotherapy. These results suggest a model of drug-resistance acquisition involving both intrinsic and acquired modes of evolution. According to the newly proposed model, drug resistance-associated CNAs are acquired in rare tumor clones during several short evolutionary bursts at the earliest stages of tumor progression and then subject to gradual evolution. Following anti-tumor therapies, the selective pressure will result in two fates for tumor cells: clonal extinction and persistence, during which the pre-existing rare drug-resistant tumor clones will persist and become the major clones. The transcriptional programs of the persisting clones will converge on a few common pathways associated with the therapy-resistance phenotypes. Both genomic mutations and transcriptional reprogramming could be relevant in understanding therapy resistance as they might exert different modes of evolution for changes at individual levels. It remains unclear how different mechanisms coordinate with one another; therefore, more powerful technologies, such as single-cell multi-omics, are needed to address these questions.

## Dissecting the tumor microenvironment to understand cancer immune evasion and metastasis

The tumor microenvironment represents all components of a solid tumor that are not cancer cells. Besides the genetic and non-genetic heterogeneity among tumor clones, heterogeneity among tumor-infiltrating stromal and immune cells in the microenvironment also plays vital roles in tumor growth, angiogenesis, immune evasion, metastasis, and responses to various therapies. With bulk DNA sequencing, the genomes of these cells in the microenvironment are indistinguishable from those of normal tissues and thus often interfere with the detection of tumor CNAs and point mutations by altering tumor purity. With bulk RNA sequencing, the mRNAs of these cells are intermingled with those of tumor cells, which makes it difficult to untangle the expression signals by tumor cells from those by microenvironment cells. The variable compositions of tumor microenvironment often become ‘dark matter’ that confounds subsequent analyses. Although pathway analysis may indicate major types of infiltrated cells, the results are not sufficiently detailed to provide insights into the underlying mechanisms of tumor phenotypes. Computational deconvolution analysis can infer tumor-infiltrating cell types based on tumor bulk RNA-seq data [128–130]. However, these algorithms are limited by the availability of gene signatures specific to individual cell types and the collinearity among gene signature profiles.

The majority of these limitations are overcome by single-cell sequencing. With scRNA-seq, the immune landscapes of melanoma [47], glioblastoma [176], breast [52, 55, 56], head and neck [48], colorectal [50], liver [49], kidney, [54, 58] and lung [53, 57, 59] cancers have been depicted at unprecedented resolution. New immune cell subtypes with distinct functions or states have been identified, and genes specifically expressed in rare immune cells have been linked to tumor immune evasion. For example, results from a recent single cell study of lung cancers by 10X Genomics [59] revealed that tumor-enriched B cells can be further grouped into six clusters, of which two follicular B cell clusters are characterized by the high expression of CD20, CXCR4, and HLA-DRs. By contrast, two plasma B-cell clusters express immunoglobulin gamma and the remaining two mucosa-associated lymphoid tissue-derived B-cell clusters have immunoglobulins A and M and JCHAIN as signature molecules. Subtypes of macrophages were also depicted by mass cytometry [53]. In particular, T cells, which specifically recognize tumor neoantigens and kill cancer cells in a targeted way, have been in the spotlight of single cell interrogation of several cancer types [49, 55, 57]. Tissue-resident T-cell subsets are found in liver, lung, and breast tumors, with lower T-cell exhaustion levels associated with better prognosis [49, 55, 57]. Immunotherapies that reinvigorate cytotoxic T cells via immune checkpoint blockade or adoptively transfer neoantigen-specific T cells are therapeutically effective in multiple cancer types [177]. Specific T-cell clusters with suppressive functions in treatment-naïve tumors and T-cell clusters that respond to immunotherapies have been identified [47, 49, 178, 179]. Signature genes of these T-cell clusters, e.g., *LAYN* identified in exhausted CD8^+^ T cells and regulatory T cells of liver cancer, can provide attractive biomarkers to predict patient responses to cancer immunotherapies and potentially serve as new candidate targets for further investigation. Nevertheless, accompanying these great achievements, single-cell studies of tumor microenvironment are limited in their depictions of spatial, temporal, and interactive characteristics among cancer and immune cells.

Besides the immune cells themselves, cancer-associated fibroblasts (CAFs) also play crucial roles in cancer immune evasion and metastasis. Heterogeneity of CAFs in various cancer types via scRNA-seq has been shown in several studies [47, 48, 50, 59]. In lung cancer studies by 10X Genomics [59], five distinct types of tumor-resident fibroblasts were identified that expressed unique repertoires of collagens and other extracellular matrix molecules. In colorectal cancers profiled by SMART-seq2 [50], two distinct subtypes of CAFs were identified, one of which was enriched for epithelial–mesenchymal transition (EMT)-related genes, which is consistent with results from the lung cancer study [59]. The heterogeneity of CAFs of these cancer types was consistent with results from earlier studies in metastatic melanoma and head and neck cancer, in which the potential functions of CAF subclusters were indicated [47, 48]. Interestingly, a specific subcluster of CAFs that exclusively expressed multiple complement factors, including C1S, C1R, C3, C4A, CFB, and C1NH (SERPING1), correlated with T-cell infiltration based on data analysis from the Cancer Genome Atlas project [47]. Although the correlation cannot imply causality, the cellular and molecular mechanisms of T-cell recruitment by CAFs should be studied. Furthermore, certain CAFs observed in a head and neck cancer single-cell study were found to co-localize with malignant cells highly expressing a p-EMT (partial EMT) gene program that is correlated with metastasis [48]. The co-localization was supported by numerous ligand–receptor interactions between CAFs and the corresponding malignant cells, thus providing new clues for the underlying mechanisms of tumor invasion. The dynamic nature of CAF gene expression certainly deserves further exploration.

## Outlook of single-cell sequencing in cancer research

**Fig. 3:**
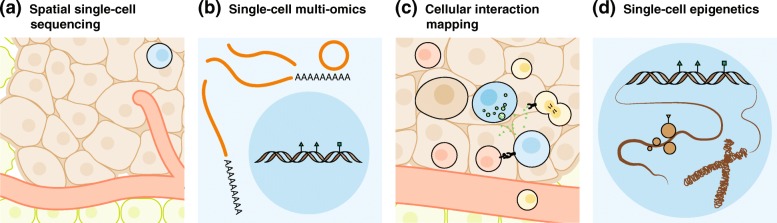
Potential applications of single-cell sequencing technologies in cancer research. **a** Spatial single-cell sequencing. Integration of single-cell sequencing technologies with spatial information of cells to analyze the spatial architecture of tumors. This technique is not yet widely used but is important for cancer biology and treatment. **b** Single-cell multi-omics. Interrogation of the cellular interaction network within tumors by single-cell sequencing. The very recent development of ProximID, which maps physical cellular interaction networks via single-cell RNA-seq without prior knowledge of component cell types, has proved the principles of single-cell multi-omics [194] and provides great promise for cancer research. **c** Cellular interaction mapping. Application of single-cell multi-omics techniques to resolve both the somatic mutations and gene expression, which will allow the investigation of immunogenicity of single cancer cells. **d** Single-cell epigenetics. Techniques to resolve the heterogeneity of cancer cells and tumor-infiltrating immune cells, which may provide new insights into the regulatory mechanisms within tumors and new drug targets to modulate tumor progression

Single-cell epigenomic technologies are maturing and steadily making their way to cancer research [15, 68, 72, 180–190] (Fig. 3). These technologies provide various means to explore DNA methylation status, chromosome accessibility, protein binding, and high-order chromosome conformations. As single-cell epigenomic technologies depict the molecular layers connecting the genome and its functional outputs, the adaptation of single-cell epigenomic technologies to cancer research would greatly advance the understanding of regulatory mechanisms of cancer cell phenotypes and provide new therapeutic targets to combat cancers [191]. New insights may also include mechanisms of cancer cell mutagenesis as epigenomics plays key roles in chromosome stability and dynamics [192]. Single-cell epigenomic technologies may also help investigate the regulatory mechanisms that shape tumor-infiltrating cells, and thus help in advancing the development of therapies that target the tumor microenvironment.

Despite its exciting prospects, single-cell sequencing still faces notable technical challenges that limit the release of its full power in cancer research and clinical applications. For example, the single layer–omics technology generally only gives a snapshot of the state of tested cells. Thorough understanding of the functions of individual cells often requires comprehensive molecular information that covers all layers from the nucleus to extracellular matrix, and includes genomes, epigenomes, chromosome confirmation, transcriptomes, proteomes, metabolomes, and interactomes (Fig. 3). Comprehensive information is important for cancer studies because of the great genomic and phonemic heterogeneity of cancer cells. Single-cell multi-omics technologies [32, 76–79, 124, 187, 193] have proved feasible but these methods are still in the infant phase of development, limited by low coverage, throughput, and automation levels. Wide application of such technologies in cancer research and clinics requires more effort to conquer the aforementioned challenges. CITE-seq has been used to simultaneously profile mRNA levels and the abundance of a set of selected proteins of cancer samples [80]. Furthermore, SUPeR-seq allows simultaneous measuring of linear and circular RNA levels within the same single cancer cell and associated cells [124], and G&T-seq provides both genomic and transcriptomic information of a given cell [76]. scTrio-seq has been used to obtain epigenomic, genomic, and transcriptomic information of the same cancer cell [32].

Future challenges will include circumventing the loss of spatial information of tested single cells during the dissociation step. Tumor ecosystems are highly organized and dynamic; therefore, the spatial positions of various cancer cells and the tumor microenvironment cells and their interactions may play pivotal roles during cancer progression, metastasis, immune evasion, and the development of therapeutic resistance (Fig. 3). Integration of imaging techniques with single-cell sequencing have made meaningful progress in this area. By recording the spatial information of single cells or important ‘anchor genes’ via FISH, smFISH, immunohistochemistry, laser capture microdissection, laser scanning microscopy, or in situ sequencing, the spatial structure of single cells can be experimentally recorded or computationally reconstructed [21, 87–91, 132, 138, 143, 149], thereby shedding light on the spatial heterogeneity of tumor ecosystems. The recently developed NICHE-seq technology [89] allows isolation of immune cells in a specifically prescribed niche of model animals for single-cell sequencing, which provides a powerful tool to explore tumor immunology in animal models. However, the wider application of NICHE-seq to clinical samples will take time, because two-photon laser scanning microscopy requires the targeted cells to be optically labeled, which at present is only possible on model animals. ProximID maps the cellular interaction network of tissues and could be used for spatial position mapping to cellular physical networks [194] to show how cancer cells interplay with the tumor microenvironment (Fig. 3). ProximID dissects tissues into doublets or triplets to capture the physical interactions among cells and determines cellular identities via scRNA-seq. ProximID provides great promise for cellular interaction and spatial position mapping, as shown by the recently proposed paired-cell sequencing method that adopts a similar strategy [195]; however, cellular throughput is still modest at present. A newer version of ProximID parallels the microdissection of doublets and triplets with single cell identity determination, and improves the throughput at the expense of accuracy of cell identity assignment. Overall, creative technological advances in the basic research field have recently emerged in quick succession. Despite obvious pros and cons, they provide exciting new tools to interrogate human cancers at the single-cell level.

Furthermore, the development of new computational and analytical tools is often lagging behind corresponding experimental methods. The new single-cell sequencing data, with added new dimensions or features, often violate the analytical assumptions of bulk sequencing studies, which makes existing analytical tools obsolete or underpowered. For example, the data structure of single-cell sequencing of cancers requires the application of tensors to depict the gene-by-cell-by-sample relationships, whereas the bulk sequencing data can be sufficiently encapsulated by gene-by-sample matrices. Analytical tools currently available are generally designed for matrix-based data structure. Reduction of dimensionality from tensors to matrices is currently needed to use the available bioinformatics tools to analyze either gene-by-cell, gene-by-sample, or cell-by-sample relationships. Tools for simultaneous analysis of gene–cell–sample relationships are urgently needed. The ever-increasing data size of single-cell sequencing studies also requires more robust computational powers. Down-sampling is often applied to reduce data size so that the dataset can be analyzed. Computational algorithms that can handle large single-cell sequencing datasets while simultaneously maintaining similar analytical performance are needed. The spatial single-cell RNA sequencing technique also generates unprecedented data type, for which two new algorithms have been proposed recently [196, 197], allowing analysis of the spatial variance of cancers. Computational development specifically for single-cell data will likely be the field to watch in the next few years, because there are many unresolved yet important issues. It is hoped that bioinformatics of single-cell analysis will catch up with the rapid technology development and the ever-expanding appetite for new data in the cancer research field.

## Potential applications of single-cell sequencing in the clinic

Single-cell technologies use limited input materials to resolve tumor heterogeneity and so have great potential in the cancer clinic for diagnosis, prognosis, early detection, risk assessment, progress monitoring, and therapy response prediction. Single cancerous cells can be isolated from blood samples in early stages of cancer genesis [161, 170, 172], which enables early detection and assessment of cancers [198, 199]. If a set of known driver mutations are observed independently in multiple single cancer cells, clonal expansion of cancerous cells is inferred. Additional diagnostic tests are then combined to validate the inference, and further monitoring or treatments may be needed. For diagnosed cancer patients, single-cell sequencing can reveal clonal and subclonal information of their tumor lesions with respect to their genomic and transcriptomic characteristics, upon which clinicians can determine the most suitable therapies [200]. With longitudinal sampling of CTCs or DTCs (disseminated tumor cells), single-cell sequencing also allows the monitoring of patient responses to the prescribed therapies [31, 171, 201]. The resulting genomic and transcriptomic information can be used to examine the selective pressure of drugs to various cancer clones and alert the emergence or expansion of drug-resistance cancer clones [20]. The non-invasive nature of CTC or DTC isolation also greatly reduces the inherent risks of core biopsy directly at the tumor site. Single-cell sequencing data potentially provide metrics beyond conventional genomic mutation data or gene expression data for prognosis analysis. For example, various indices for tumor heterogeneity could be designed to predict responses to therapies, probability of metastasis, disease-free periods, and overall survival [147, 202–205].

## Conclusions

Since its inception, single-cell sequencing has revolutionized cancer research. The pioneering studies have covered the development and applications of single-cell DNA and RNA sequencing to address a wide range of topics such as intra-tumor heterogeneity of primary tumors, roles of CTCs and DTCs during metastasis, evolution of therapy resistance, and the characteristics of tumor microenvironments. Many novel biological insights have been obtained, and the revolution is just starting. Improvement of existing single-cell sequencing technologies, emergence of new techniques, and the integration of single-cell sequencing with other experimental protocols have provided powerful toolsets to understand many of the remaining mysteries of cancers. Single-cell epigenomics, multi-omics, and spatial single-cell sequencing technologies are some of the major directions of single-cell sequencing technologies that will bring the second wave of revolutions of cancer research.

## Abbreviations

CAF: Cancer-associated fibroblast
CNA: Copy number alteration
CTC: Circulating tumor cells
DTC: Disseminated tumor cells
EMT: Epithelial–mesenchymal transition
FISH: Fluorescence in situ hybridization
scRNA-seq: Single-cell RNA sequencing
sm-FISH: Single molecule fluorescence in situ hybridization
SNV: Single nucleotide variant
